# Recent advances in understanding the molecular mechanisms of the development and function of Th17 cells

**DOI:** 10.1111/gtc.12039

**Published:** 2013-02-05

**Authors:** Yutaka Kurebayashi, Shigenori Nagai, Ai Ikejiri, Shigeo Koyasu

**Affiliations:** 1 Department of Microbiology and Immunology, Keio University School of MedicineShinjuku-ku, Tokyo, 160-8582, Japan; 2 Core Research for Evolutional Science and Technology (CREST), Japan Science and Technology (JST)Tokyo, 102-0075, Japan; 3 Department of Immunology, National Institute of Infectious DiseasesShinjuku-ku, Tokyo, 162-8640, Japan; 4 Laboratory for Immune Cell System, RIKEN Research Center for Allergy and Immunology (RCAI)Yokohama, Kanagawa, 230-0045, Japan; 5 Research Center for Science Systems, Japan Society for the Program of Science (JSPS)Tokyo, 102-8472, Japan

## Abstract

IL-17-producing T helper (Th17) cells comprise a distinct Th subset involved in epithelial cell- and neutrophil-mediated immune responses against extracellular microbes. At the same time, Th17 cells play significant roles in the development of autoimmune diseases including rheumatoid arthritis and multiple sclerosis. Since the identification of Th17 cells approximately a decade ago, the molecular mechanisms of their differentiation have been intensively studied and a number of signaling cascades and transcription factors have been shown to be involved. Here, we review the current knowledge regarding the function of Th17 cells *in vivo* as well as several key concepts for the molecular mechanisms of Th17 differentiation. We also discuss the emerging roles of phosphoinositide 3-kinase (PI3K), mammalian target of rapamycin complex 1 (mTORC1) and hypoxia-inducible factor 1 (HIF-1) in the differentiation of Th17 cells.

## Introduction

Immune systems are generally divided into the innate and adaptive arms, and CD4^+^ T helper (Th) cells are indispensable for initiating the latter reaction. Th cells are subdivided into several subsets with distinct functions: T helper type 1 (Th1), T helper type 2 (Th2), IL-17-producing T helper (Th17), IL-9-producing T helper (Th9), or follicular T helper (Tfh) cells (Mosmann & Coffman 1989; Ouyang *et al*. 2008; Veldhoen *et al*. 2008b; Fazilleau *et al*. 2009). Th1 cells produce IFN-γ, activate macrophages and support granuloma formation as observed in *Mycobacterium tuberculosis* infection, whereas Th2 cells produce IL-4, IL-5 and IL-13, assist in the generation of IgE-producing plasma cells from naïve B cells, activate mast cells and eosinophils and support antihelminth immunity as well as allergic reactions. Th9 cells were recently identified as an IL-9-producing subtype possibly contributing to the induction of intestinal mucosal mast cells. Tfh cells produce IL-21 and provide B cell help in the lymph node germinal centers. There are also other CD4^+^ T-cell subsets with regulatory roles such as thymus-derived naturally occurring regulatory T cells (nTregs), inducible regulatory T cells (iTregs) and regulatory type 1 cells (Tr1) (Roncarolo *et al*. 2006; Sakaguchi *et al*. 2008).

Th17 cells are characterized by the production of IL-17A, IL-17F and IL-22. These Th17 cytokines induce the expression of numerous chemokines and antimicrobial peptides in epithelial cells and fibroblasts, which are important for the neutrophil-mediated immune reactions against extracellular microbes (Ouyang *et al*. 2008). Th17 cells are also a focus of attention because of their roles in the pathogenesis of various autoimmune diseases including rheumatoid arthritis (RA) and multiple sclerosis (MS). Since their identification, the molecular mechanisms of Th17 differentiation have been intensively studied, and numerous intracellular signaling cascades and transcriptional factors have now been identified. Recently, phosphoinositide 3-kinase (PI3K), mammalian target of rapamycin complex 1 (mTORC1) and hypoxia-inducible factor-1 (HIF-1) have been shown to regulate Th17 differentiation positively as well (Dang *et al*. 2011; Delgoffe *et al*. 2011; Ikejiri *et al*. 2011; Shi *et al*. 2011; Kurebayashi *et al*. 2012). Herein, we summarize the molecular mechanisms governing the Th17 differentiation in light of recent important findings. Although some of these findings still require further confirmation *in vivo*, understanding of these molecular complexities in Th17 differentiation may provide us the basis to define more clearly how these cells are generated and contribute to the host defense and to the development of autoimmune diseases.

## Role of Th17 cells in bacterial and fungal infection

IL-17A was first identified as murine cytotoxic T lymphocyte–associated antigen-8 (mCTLA8) (Rouvier *et al*. 1993), which shows 57% homology with the amino acid sequence of the open reading frame 13 (ORF13) of T lymphotrophic virus *Herpesvirus saimiri*. Subsequently, IL-17A receptor (IL-17R) was cloned (Yao *et al*. 1995). Further studies expanded the IL-17 protein family from IL-17A to IL-17F (Kolls & Linden 2004), and Th17 cells were characterized as one of the major sources of IL-17A and IL-17F (Aggarwal *et al*. 2003; Bettelli & Kuchroo 2005; Langrish *et al*. 2005).

Early studies in murine infection models have established IL-17A derived from Th17 cells and IL-17-producing CD8^+^ T (Tc17) cells as important regulators of host defense against extracellular bacteria such as *Klebsiella pneumoniae* (Ye *et al*. 2001; Happel *et al*. 2005). In these models, IL-17A stimulates epithelial cells and fibroblasts to produce inflammatory mediators such as IL-6, macrophage inflammatory protein-2 (MIP-2), granulocyte colony-stimulating factor (G-CSF), prostaglandin E2 (PGE2) and several CXC chemokines, thus promoting granulopoiesis and neutrophil recruitment required for host defense against extracellular bacteria (Fossiez *et al*. 1996; Khader *et al*. 2009). IL-17A, IL-17F and IL-22 also induce the production of antimicrobial peptides such as β-defensin-2, S100 proteins and lipocalin-2 from mucosal (e.g. pulmonary and intestinal) epithelial cells (Khader *et al*. 2009). Later, studies indicate γδ T cells and invariant natural killer T (iNKT) cells as other major sources of IL-17A during *K. pneumoniae* infection (Price *et al*. 2012). In the intestinal mucosa as well, CD8^+^ T cells, γδ T cells, natural killer (NK) cells and innate lymphoid cells are also important producers of Th17 cytokines (Maynard *et al*. 2012); therefore, not only Th17 cells, but also other constellation of innate and adaptive sources of IL-17A, IL-17F and IL-22 are required for the effective host defense against extracellular bacterial infections. Host defense mechanisms against *Citrobacter rodentium* and *Staphylococcus aureus* also depend on Th17 cytokines (Ishigame *et al*. 2009; Khader *et al*. 2009).

Th17 cells are also required for the host defense against fungal infections depending on the species and the sites of infections. αβ T cells and their production of IL-17A are pivotal in the host defense against oral infection with *Candida albicans*, whereas Th1-related cytokine IL-12 prevents its systemic dissemination (Conti *et al*. 2009). In *Aspergillus fumigatus* infection, the host defense mainly relies on Th1 responses rather than Th17 responses (Romani 2011). In humans, patients with autosomal dominant hyper IgE syndrome (HIES) carry mutations in *Stat3*, presenting impaired Th17 differentiation and increased susceptibility to candidal and staphylococcal infections (Milner *et al*. 2008). Autosomal recessive IL-17RA deficiency and autosomal dominant IL-17F deficiency also lead to chronic mucocutaneous candidiasis (CMC) with *S. aureus* dermatitis (Puel *et al*. 2011). Similarly, those who develop autoantibodies against IL-17A, IL-17F and IL-22 suffer CMC (Kisand *et al*. 2010; Puel *et al*. 2010), which strikingly contrasts to the patients with anti-IFNγ autoantibody, who present disseminated nontuberous mycobacterial infections (Browne *et al*. 2012).

Additional studies revealed diverse roles of IL-17A in Th1-mediated immunity and B-cell biology. For instance, IL-17A directly activates macrophages and dendritic cells to produce various cytokines including IL-12, which enhances Th1 immunity and host defense against infection by the intracellular bacteria, *Francisella tularensis* (Lin *et al*. 2009). IL-17R is also expressed on B cells, and IL-17A promotes germinal center formation and B-cell survival and proliferation by activating NF-κB pathways (Hsu *et al*. 2008; Doreau *et al*. 2009; Xie *et al*. 2010). Recent findings show that ectopic lymph node formation in the lung upon infections requires IL-17A derived from CD4^+^ T cells (Rangel-Moreno *et al*. 2011).

## Role of Th17 cells in the pathogenesis of autoimmunity

IL-17A also promotes the development of autoimmune diseases (Ouyang *et al*. 2008). In both murine collagen-induced arthritis (CIA) model and IL-1RA^-/-^ arthritis model, deletion of IL-17A or p19 subunit of Th17-related cytokine IL-23 significantly attenuates the severity of disease (Murphy *et al*. 2003; Nakae *et al*. 2003a,b). IL-17A activates osteoblasts and synovial fibroblasts to express RANKL, which is required for osteoclast differentiation (Kotake *et al*. 1999; Sato *et al*. 2006), and thus, IL-17A contributes to bone destruction in RA. Experimental autoimmune encephalomyelitis (EAE) is an animal disease model of human MS, an autoimmune disease in central nervous system (CNS), and the deletion of IL-23 p19 subunit confers resistance to murine EAE (Cua *et al*. 2003). Th17 cell-derived IL-17A and IL-22 have been shown to act on blood–brain barrier (BBB) endothelial cells and disrupt the structure of BBB, enabling the infiltration of pathogenic Th17 cells into CNS lesion, suggesting the pathogenic role of Th17 cells. However, accumulating evidence shows that the deletion of IL-17A, IL-17F and IL-22 leads to only limited effects on the severity of EAE (Komiyama *et al*. 2006; Haak *et al*. 2009). Instead, RORγt-dependent expression of GM-CSF from Th cells activated by IL-23 has a pivotal role in the pathogenesis; Th cell-derived GM-CSF augments the infiltration of CD45^hi^CD11b^+^ myeloid cells into CNS and contributes to the development of EAE (Codarri *et al*. 2011). In murine colitis models, IL-17A production from CD4^+^ T cells is protective because IL-17A directly suppresses the development of colitogenic Th1 cells via IL-17R expressed on activated CD4^+^ T cells (O'Connor *et al*. 2009). In contrast, as in other autoimmune disease models, IL-23 accelerates the severity of murine colitis (Ahern *et al*. 2010). It is generally believed that IL-17-producing cells are protective but that IL-17/IFN-γ double producers are pathogenic and IL-23 accelerates the generation of double producers (Ahern *et al*. 2010; Hirota *et al*. 2011).

IL-17A is also required for Th2-mediated OVA-induced asthmatic reactions in mice (Nakae *et al*. 2002). Clinically, two different processes coexist in human asthma: corticosteroid-sensitive Th2 inflammatory mechanisms and corticosteroid-resistant airway remodeling characterized by subepithelial fibrosis and increased smooth muscle volume (Hackett 2012). Th17 cells are shown to contribute to the latter process in murine OVA-induced asthma model (Zhao *et al*. 2013). Bronchoalveolar lavage fluid (BALF) from asthma patients contains high amounts of IL-4/IL-17A double-positive CD4^+^ T cells (Wang *et al*. 2010), and moderate-to-severe human asthma patients have more IL-17A-positive cells in bronchial submucosa than mild asthma patients (Chakir *et al*. 2003). These observations indicate the role of Th17 cells in airway remodeling in asthma patients.

## Developmental regulation of Th17 cells by cytokines and environmental factors

**Figure 1:**
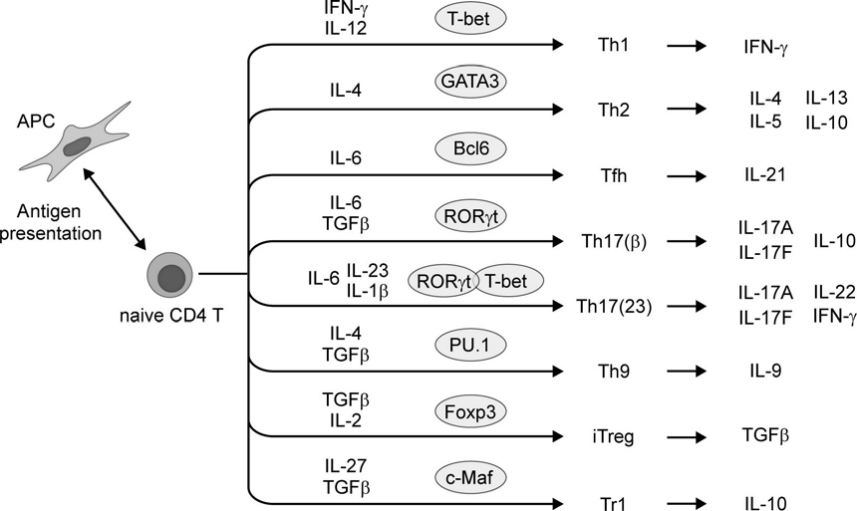
Cytokines and transcription factors required for Th differentiation. Upon antigen presentation, IL-12 and IFN-γ are required for Th1 differentiation, IL-4 for Th2 differentiation, IL-6 for Tfh differentiation, IL-6 and TGF-β for Th17(β) differentiation, IL-6, IL-23 and IL-1β for Th17(23) differentiation, IL-4 and TGF-β for Th9 differentiation, TGF-β and IL-2 for iTreg differentiation and IL-27 and TGF-β for Tr1 differentiation. These cytokines enable CD4^+^ T cells to express critical transcription factors that direct for the differentiation of each Th subset: T-bet for Th1 differentiation, GATA3 for Th2 differentiation, Bcl-6 for Tfh differentiation, RORγt for Th17(β) and Th17(23) differentiation, PU.1 for Th9 differentiation, Foxp3 for iTreg differentiation, and c-Maf for Tr1 differentiation. Th17(23) differentiation is characterized by the co-expression of RORγt and T-bet. Cytokines produced by each Th cell lineage are also indicated.

Upon antigen presentation by antigen-presenting cells (APCs), naïve CD4^+^ T cells differentiate into any Th subset based on the cytokine milieu produced by the presenting APCs and surrounding mesenchymal cells (Dong 2006; also see Fig. 1). For example, development of Th1 cells requires IL-12 and IFN-γ whereas that of Th2 cells requires IL-4. Each cytokine required for the differentiation of one Th subset negatively regulates the differentiation of the other, for example, IFN-γ and IL-12 inhibit Th2 differentiation and IL-4 inhibits Th1 differentiation.

The cytokines initially linked to Th17 differentiation were IL-6 and IL-23 (Infante-Duarte *et al*. 2000; Aggarwal *et al*. 2003; Langrish *et al*. 2005). Later, TGF-β together with IL-6 was shown to initiate Th17 differentiation both *in vitro* and *in vivo* (Mangan *et al*. 2006; Veldhoen *et al*. 2006). It is now widely accepted that Th17 cells can be divided into two different subsets according to cytokine requirements for their differentiation and the expression profiles of cytokines and chemokines (Fig. 1). One is conventional Th17 (Th17(β)) cells differentiated by IL-6 and TGF-β, which express higher IL-10, CCL20 and CXCR6 in addition to IL-17A and IL-17F. The other is Th17(23) cells differentiated by IL-6, IL-23 and IL-1β without exogenous TGF-β, which are characterized by the expression of higher IL-22, CCL9 and CXCR3 (Ghoreschi *et al*. 2010). IL-21 is also indispensable for the development and expansion of both Th17(β) and Th17(23) cells *in vivo* both in humans and mice (Korn *et al*. 2007; Yang *et al*. 2008a), and IL-1β augments the differentiation of not only Th17(23) cells but also Th17(β) cells both *in vitro* and *in vivo* (Sutton *et al*. 2006; Gulen *et al*. 2010; Shaw *et al*. 2012). Accumulating evidence shows that Th17(23) cells possess higher pathogenic ability in autoimmune models (Ghoreschi *et al*. 2010), and IL-23 also has a pivotal role in the conversion of IL-17A single-positive Th17 cells into IL-17A/IFN-γ double producers *in vivo* (Hirota *et al*. 2011). Similar demarcation is observed in human memory Th17 cells, in which *C. albicans*-specific Th17 cells co-express higher IFN-γ than *S. aureus*-specific Th17 cells. The increased co-expression of IFN-γ is dependent on IL-1β, and only *S. aureus*-specific Th17 cells exhibit the ability for IL-10 expression upon restimulation (Zielinski *et al*. 2012).

In terms of cellular sources of these Th17-inducing cytokines, dendritic cells (DCs) produce IL-1β, IL-6 and IL-23 during antigen presentation, and it has recently been shown that Th17 cells support their own differentiation by producing TGF-β in an autocrine manner (Gutcher *et al*. 2011); however, the exact source of TGF-β in the initial differentiation of Th17 cells is still unclear. As with the opposing regulation between Th1 and Th2 differentiation, cytokines required for Th1 and Th2 differentiation (IFN-γ, IL-12 and IL-4) inhibit Th17 differentiation (Infante-Duarte *et al*. 2000; Harrington *et al*. 2005). Th1 and Th2 cells also inhibit Th17 differentiation through IFN-γ and IL-4, respectively. Th1 and Th2 cells expand with the help of IL-2 in an autocrine manner, but IL-2 severely dampens Th17 differentiation (Laurence *et al*. 2007). IL-27, which is also produced by APCs and induces the development of IL-10-producing Tr1 cells, inhibits Th17 differentiation and GM-CSF production, thus negatively regulating the severity of EAE (Stumhofer *et al*. 2006, 2007; Codarri *et al*. 2011). Notably, IL-6 in the absence of TGF-β initiates Tfh differentiation (Fazilleau *et al*. 2009) and TGF-β without IL-6 results in iTreg differentiation (Sakaguchi *et al*. 2008). It was in a way a surprise that TGF-β is required for the differentiation of Th17 cells in the presence of inflammatory cytokine IL-6 because TGF-β, an indispensable cytokine for the generation of iTreg cells, had been recognized as an anti-inflammatory cytokine with a regulatory nature. Hence, the balance between the production of pro- and anti-inflammatory cytokines from APCs is a key modulator of the development of each CD4^+^ T-cell lineage including Th17(β), Th17(23), Tfh and iTreg cells, which is determined as a result of the complex intracellular signaling interactions in APCs generated by the recognition of various antigens exposed on pathogens as described in detail elsewhere (Kawai & Akira 2011).

In a steady state, major population of Th17 cells harbor in the intestinal mucosa, whose development is largely dependent on the colonization of commensal microbiota (Ivanov *et al*. 2009). There is a complex regulation to balance effector immune reactions and tolerance acquisition against commensal microbiota, and the dysregulated activation of immune system results in the development of colitis. The steady-state intestinal Th17 cells are induced and maintained by TGF-β, which is abundant in intestinal mucosa, and microbiota-induced IL-1β and IL-23 (Ghoreschi *et al*. 2010; Maynard *et al*. 2012; Shaw *et al*. 2012). Microbiota-derived ATP even activates intestinal DCs to promote Th17 differentiation (Atarashi *et al*. 2008). The negative regulation of Th17 differentiation and inflammation in the intestine is mainly achieved by all-trans-retinoic acid, which is synthesized by CD103^+^ DCs and intestinal epithelial cells from food-derived vitamin A (Maynard *et al*. 2012). Microbiota-induced expression of indoleamine-pyrrole 2,3-dioxygenase (IDO) in epithelial cells and DCs catabolizes essential amino acid tryptophan and suppresses excessive Th cell differentiation (Romani 2011).

## Th17 *vs* iTreg differentiation: RORγt *vs* Foxp3 and the role of hypoxia and HIF-1

**Figure 2:**
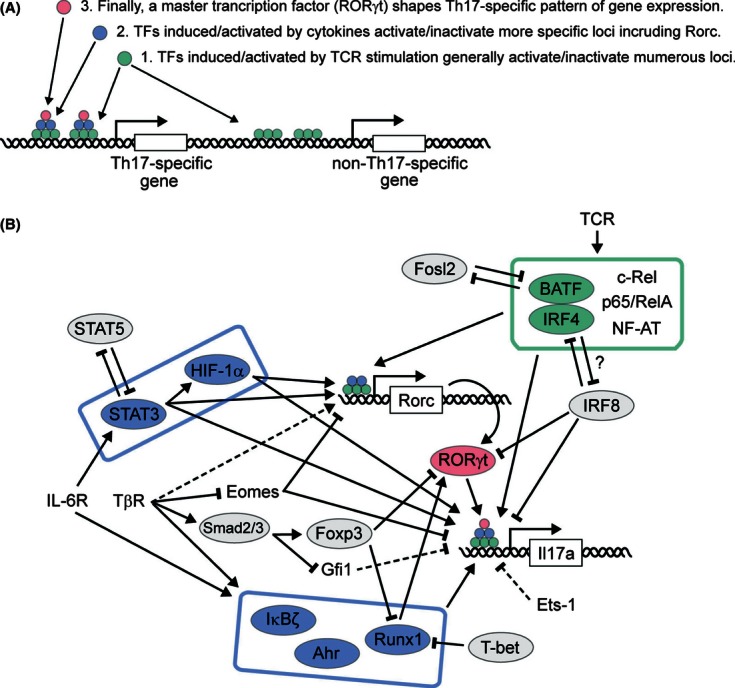
Transcription factors regulating *Rorc* and *Il17a* loci expression. (A) Schematic overview of the stepwise regulation of Th17-related loci expression. TCR-induced/TCR-activated transcription factors (TFs, green) bind to and activate/inactivate numerous Th17-specific and non-Th17-specific loci. Next, cytokine-induced/cytokine-activated TFs (blue) activate/inactivate more limited numbers of loci including a critical transcription factor RORγt (red), outlining the Th17-specific pattern of gene expression. Finally, a master transcription factor RORγt determines Th17-specific pattern of gene expression. (B) Schematic description of transcription factors regulating Th17 differentiation. BATF, IRF4, c-Rel, p65/RelA and NF-AT are TCR-induced/TCR-activated TFs generally activating/inactivating numerous loci (green box). Fosl2 and IRF8 compete with BATF and IRF4 for their target loci, respectively, and negatively regulate Th17 differentiation. Next, cytokine-induced/cytokine-activated TFs such as STAT3, HIF-1α, Runx1, IκBζ and Ahr outline the Th17-specific pattern of gene expression (blue box). STAT5 competes with STAT3 for their target loci and decreases Th17 differentiation. TGF-β-induced activation of Smad2/3 induces Foxp3 expression, which directly interacts with and inhibits the function of RORγt. Foxp3 also interacts with Runx1 and abrogates the positive interaction of Runx1 with RORγt. T-bet also directly interacts with Runx1 and interrupts its positive interaction with RORγt. TGF-β signaling decreases the expression of Eomes, a negative regulator of *Rorc* and *Il17a* expression. Gfi-1 and Ets-1 are negative regulators of Th17 differentiation without known functional mechanisms. The expression of Gfi-1 is also down-regulated by TGF-β signaling (see also Table 1).

The differentiation of each Th cell subset defined by the local cytokine milieu is achieved by the expression of specific transcription factors (Dong 2006; also see Fig. 1): T-bet in Th1 differentiation, GATA3 in Th2 differentiation, PU.1 in Th9 differentiation (Chang *et al*. 2010), or Bcl6 in Tfh differentiation (Fazilleau *et al*. 2009). Tregs are characterized by their expression of Foxp3 (Sakaguchi *et al*. 2008), and IL-10 production from Tr1 cells depends on c-Maf (Pot *et al*. 2009; Apetoh *et al*. 2010). In Th17 cells, RORγt, encoded by *Rorc* gene, is a pivotal transcription factor (Fig. 2A). In fact, transduction of RORγt is sufficient to convert unpolarized CD4^+^ T cells into Th17 cells (Ivanov *et al*. 2006). A related orphan receptor RORα has also been shown to initiate Th17 differentiation together with RORγt (Yang *et al*. 2008c). It has recently been shown that the anti-arrhythmic drug digoxin and its derivatives bind to RORγt and severely impair its transcriptional activity of RORγ and Th17 differentiation (Huh *et al*. 2011). This further underscores the critical role of RORγt in Th17 differentiation.

As noted above, both pro-inflammatory Th17 and anti-inflammatory iTreg cells require TGF-β for their differentiation, and the molecular mechanism balancing Th17 versus iTreg differentiation has been intensively studied (Fig. 2B). During Th17(β) differentiation, RORγt expression is mainly induced by TGF-β (Ichiyama *et al*. 2008; Zhou *et al*. 2008) in a Smad2-/Smad3-independent manner (Takimoto *et al*. 2010) although the precise mechanisms of RORγt induction are still poorly understood. The induction of RORγt in Th17(23) cells without TGF-β signaling is more confusing, and the mechanism is largely unknown. Recently, it has been shown that TGF-β3 but not TGF-β1 (referred simply as TGF-β in this review unless otherwise indicated) is induced by IL-23 in addition to IL-6 and IL-1β, enabling RORγt expression and the development of more inflammatory Th17(23) cells (Lee *et al*. 2012). Of note, TGF-β is critical for RORγt induction but itself does not generate Th17(β) cells and instead initiates iTreg differentiation. This is because TGF-β also induces Foxp3 in a Smad2-/Smad3-dependent manner; Foxp3 interacts with RORγt and directly suppresses the transcriptional activity of RORγt, consequently blocking Th17(β) differentiation and initiating iTreg development (Ichiyama *et al*. 2008; Yang *et al*. 2008b; Zhou *et al*. 2008; also see Fig. 2B). The transcription factor Runx1 is a positive regulator of Th17 differentiation, which directly interacts with RORγt and increases its transcriptional activity (Zhang *et al*. 2008). Such Runx1-mediated increase in RORγt activity is also abrogated by the direct interaction of Foxp3 with Runx1; hence, Foxp3 directly inhibits Th17 differentiation through interaction with RORγt and indirectly through interaction with Runx1 (Zhang *et al*. 2008). The interaction of Runx1 with RORγt is also interrupted by tyrosine-phosphorylated T-bet, an important regulator of Th1 differentiation (Lazarevic *et al*. 2011), which is also induced in Th17(23) cells and actually limits IL-17A expression (Ghoreschi *et al*. 2010). The expression of Foxp3 is suppressed by addition of IL-6 and IL-21 in a STAT3-dependent manner, which enables RORγt to initiate Th17 differentiation (Ichiyama *et al*. 2008; Yang *et al*. 2008b; Zhou *et al*. 2008). Therefore, IL-6 and IL-21 serve as key cytokines in initiating Th17-mediated inflammatory reactions in the presence of TGF-β.

The mechanism how STAT3 down-regulates Foxp3 expression in Th17 differentiation had remained unclear, but it has recently been shown that STAT3-induced HIF-1α binds to Foxp3 and leads to the proteosomal degradation of Foxp3 during Th17 differentiation (Dang *et al*. 2011). HIF-1 is a well-characterized transcription factor induced under hypoxic conditions and consists of a heterodimer composed of an oxygen-sensitive HIF-1α subunit and a constitutively expressed HIF-1β subunit (Wang & Semenza 1993; Majmundar *et al*. 2010). Recent studies have shown that both hypoxia and HIF-1 positively and negatively regulate Th17 and iTreg differentiation, respectively, without apparent effect on Th1 or Th2 differentiation, and not only hypoxia but also IL-6 induces HIF-1α expression during Th17 differentiation via STAT3 activation (Dang *et al*. 2011; Ikejiri *et al*. 2011; Shi *et al*. 2011). Oxygen-dependent degradation of HIF-1α is mediated by E3 ubiquitin ligases, which contain von Hippel–Lindau tumor suppressor protein (pVHL), and the deletion of pVHL results in increased Th17 differentiation (Ikejiri *et al*. 2011). In addition to the proteosomal degradation of Foxp3 by HIF-1α, it also promotes Th17 differentiation via several other mechanisms. First, HIF-1 binds to hypoxia response elements (HREs) located in the proximal region of the *Rorc* locus and enhances its expression. HIF-1 also forms a complex with RORγt and recruits p300 to the *Il17a*, *Il17f* and *Il23r* loci. In addition, Shi *et al*. (2011) have shown that during Th17 differentiation, HIF-1 positively controls the glycolysis required for the rapid T-cell expansion after TCR stimulation.

In addition to inducing HIF-1α and down-regulating Foxp3 expression, IL-6 and STAT3 signaling have further important roles during Th17 differentiation. For example, STAT3 directly binds to and activates the expression of loci encoding Th17-related molecules and cytokines (Chen *et al*. 2006; Yang *et al*. 2011b; Ciofani *et al*. 2012), and interaction of STAT3 to *Il17a* and *Il17f* loci is directly competed by STAT5 (Yang *et al*. 2011b), which explains the negative regulatory role of IL-2 on Th17 differentiation. Intriguingly, even a combination of TGF-β treatment and the expression of a constitutively active form of STAT3 is insufficient for the full differentiation of Th17 cells (Zhou *et al*. 2007), indicating the presence of a yet to be identified factor for Th17 differentiation.

## Regulation of Th17 differentiation by other transcription factors

In addition to Foxp3, Runx1, STAT3 and HIF-1, the expression of RORγt and Th17 cytokines is also regulated by numerous other transcription factors. These transcription factors are induced in a stepwise fashion and can be classified into following five categories according to the order of their expression and function (Fig. 2A and Table 1): (A) those induced/activated by TCR stimulation, which contribute to general activation/inactivation of numerous loci including both Th17-specific and non-Th17-specific ones, (B) those induced/activated by cytokines, which activate/inactivate more specific loci including *Rorc*, (C) RORγt and RORα, which finally form a Th17-specific expression pattern, (D) those induced by TCR stimulation but with more specific function in Th17 differentiation, and (E) those lacking any established functional mechanisms but important in Th17 differentiation.

**Table 1:** Classification of transcription factors regulating Th17 differentiation. Transcription factors (TFs) are classified into five categories (from A to E) based on the order of expression and their functions. Groups A, B and C correspond to TFs colored in green, blue and red in Fig. 2, respectively

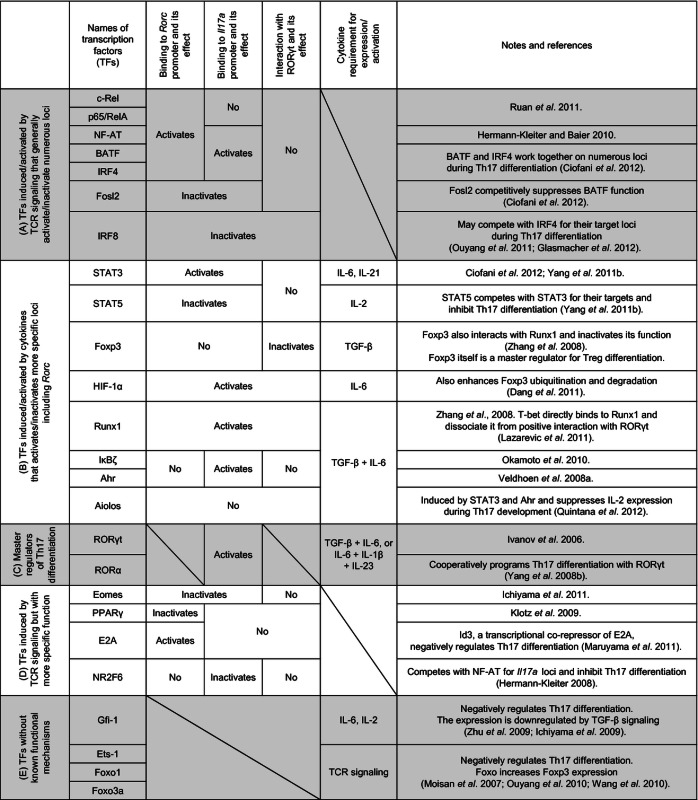

Initial signalings from TCR and costimulatory molecules induce and activate numerous transcription factors (group A in Table 1) that enable basal activation of CD4^+^ T cells required for further differentiation into each Th subset. Among the group A transcription factors, NF-κB and NF-AT family transcription factors generally activate many loci upon the activation of CD4^+^ T cells. During Th17 differentiation, RelA/p65 and c-Rel are two NF-κB family transcription factors required for the initiation of *Rorc* expression (Ruan *et al*. 2011; also see Fig. 2B). RelA/p65 and c-Rel directly bind to two putative Rel-binding site on the *Rorc* promoter and enhance RORγt expression, whereas none of NF-κB family transcription factors bind to *Il17a* promotor. RelA/p65 and c-Rel are also required for Foxp3 expression, and it forms a unique c-Rel enhanceome at *Foxp3* promotor (Ruan *et al*. 2009). T-bet expression and Th1 differentiation are c-Rel-dependent as well (Hilliard *et al*. 2002). Therefore, RelA/p65 and c-Rel require other sets of transcription factors with limited functions to achieve a Th17-specific gene expression pattern. In addition to NF-κB activation, TCR stimulation also leads to the influx of Ca^2+^ into the cytoplasm and activates the calcineurin/NF-AT pathway, which also targets loci encoding various transcription factors and cytokines (Rao *et al*. 1997). Among the Th17-related gene loci, NF-AT binds to both *Rorc* and *Il17a* promoters and activates their expression (Hermann-Kleiter & Baier 2010). A nuclear orphan receptor NR2F6 competes with NF-AT for their targets in Th17-related genes and specifically inhibits Th17 differentiation (Hermann-Kleiter *et al*. 2008).

Recent studies have highlighted BATF and IRF4 as initial activators of Th17 differentiation (Ciofani *et al*. 2012; Glasmacher *et al*. 2012). Both BATF and IRF4 are induced by TCR stimulation and indispensable for proper Th17 differentiation (Brustle *et al*. 2007; Schraml *et al*. 2009). Ciofani *et al*. (2012) showed that BATF and IRF4 have common putative *cis*-regulatory modules (pCRMs) and function together in many loci, which include the most pCRMs for STAT3 and RORγt found in *Il17a*, *Il17f*, *Il12b1*, *Il1r1* and *Rorc* loci. The binding of BATF and IRF4 to those loci increases chromatin accessibility for other transcription factors, and it is prerequisite for Th17 differentiation. Ciofani *et al*. (2012) also showed that another AP-1 family transcription factor Fosl2 competes with BATF for their target loci and works as a negative regulator of Th17 differentiation. Similarly, IRF8, a negative regulator of Th17 differentiation induced by TCR signaling (Ouyang *et al*. 2011), also shares many common pCRMs with IRF4 (Glasmacher *et al*. 2012); hence, IRF8 likely competes with IRF4 for their target loci and negatively regulates Th17 differentiation. IRF8 also directly binds to RORγt and suppresses its transcriptional activity (Ouyang *et al*. 2011). Notably, the activity of IRF4 is regulated through phosphorylation by ROCK2, a serine–threonine kinase induced during Th17 differentiation. Hence, the function of IRF4 is also partly under the control of Th17-inducing cytokines (Biswas *et al*. 2011).

Given the increased accessibility to numerous loci achieved by TCR-induced/TCR-activated transcription factors cytokine-induced/cytokine-activated transcription factors (group B of Table 1, see also Fig. 2B) form a more specific gene expression pattern for Th17 differentiation. These transcription factors include Runx1, STAT3, HIF-1, IκBζ, Ahr and Aiolos. The roles of Runx1, STAT3 and HIF-1 were mentioned above. During Th17 differentiation, the induction of IκBζ, Ahr and Aiolos requires both IL-6 and TGF-β. The induction and function of these transcription factors are mainly studied in Th17(β) cells; hence, it is still unclear whether these transcription factors are differently expressed in between Th17(β) and Th17(23) cells. IκBζ does not form complexes with RORγt but directly binds to and activates *Il17a* promoter. Among the three alternative splicing variants of IκBζ (IκBζ(L), IκBζ(S) and IκBζ(D)), IκBζ(L) and IκBζ(S) are expressed in and enhance the differentiation of Th17 cells (Okamoto *et al*. 2010). Ahr is also induced during Th17 differentiation, directly binds to *Il17a* promoter and activates the expression of IL-17A. One of the Ahr agonists 6-formylindolo(3,2-b)carbazole (FICZ) increases Th17 differentiation and exacerbates EAE, whereas Ahr antagonist resveratrol decreases the differentiation of Th17 cells (Quintana *et al*. 2008; Veldhoen *et al*. 2008a; Cui *et al*. 2011). Intriguingly, Ahr is also required for iTreg development and another Ahr agonist 2,3,7,8-tetrachlorodibenzo-p-dioxin (TCDD) accelerates iTreg development and attenuates EAE severity; hence, Ahr regulates the differentiation of Th17 and iTreg cells in a ligand-specific manner (Quintana *et al*. 2008). Ahr also binds to *Il10* promoter and enhances the expression of IL-10 together with c-Maf during Tr1 differentiation (Apetoh *et al*. 2010; Gandhi *et al*. 2010), which may also contribute to the production of IL-10 from Th17(β) cells. Aiolos is induced by STAT3 and Ahr downstream of IL-6 and TGF-β and shuts down the expression of IL-2 during Th17 differentiation (Quintana *et al*. 2012). These group A and B transcription factors briefly outline the Th17-specific gene expression patterns and induce the expression of a transcription factor RORγt (Ivanov *et al*. 2006; Ciofani *et al*. 2012). The function of RORγt is accomplished with the help of related nuclear orphan receptor RORα, which works together with RORγt and accelerates Th17 differentiation (Yang *et al*. 2008c). These group C molecules finally complete Th17-specific gene expression patterns (Fig. 2A,B).

Other transcription factors are induced by TCR stimulation but exert more specific functions in Th17 differentiation. These include Eomesodermin (Eomes), PPARγ and E2A (group D in Table 1). For instance, Eomes is induced by TCR signaling and works as a negative regulator of *Rorc* and *Il17a* expression (Ichiyama *et al*. 2011). The expression of Eomes is down-regulated by TGF-β-induced TAK1-JNK-c-Jun pathway, which explains one mechanism how Smad-independent upregulation of *Rorc* expression (Takimoto *et al*. 2010) is achieved by TGF-β during Th17 differentiation. PPARγ (Klotz *et al*. 2009) and E2A (Maruyama *et al*. 2011) are induced by TCR signaling, bind to *Rorc* promotor and inhibit and activate RORγt expression, respectively. Accordingly, antidiabetic thiazolidinediones pioglitazone, a PPARγ agonist, decreases Th17 differentiation and the severity of EAE, and Id3, a corepressor of E2A, also inhibits Th17 differentiation. Gfi1 (Ichiyama *et al*. 2009; Zhu *et al*. 2009), Ets-1 (Moisan *et al*. 2007), Foxo1 and Foxo3a (Ouyang *et al*. 2010; Wan *et al*. 2010) are also suggested as negative regulators of Th17 differentiation. However, the mechanisms by which these transcription factors work are still unclear (group E in Table 1). Regarding of iTreg differentiation, Foxo1 and Foxo3a directly bind to *Foxp3* promoter and enhance its expression (Ouyang *et al*. 2010). The expression of Gfi-1 is induced by TCR stimulation and decreased by TGF-β during Th17 differentiation (Ichiyama *et al*. 2009; Zhu *et al*. 2009).

## mTORC2-Akt-Foxo1/3a signaling in Th17 and Treg differentiation

**Figure 3:**
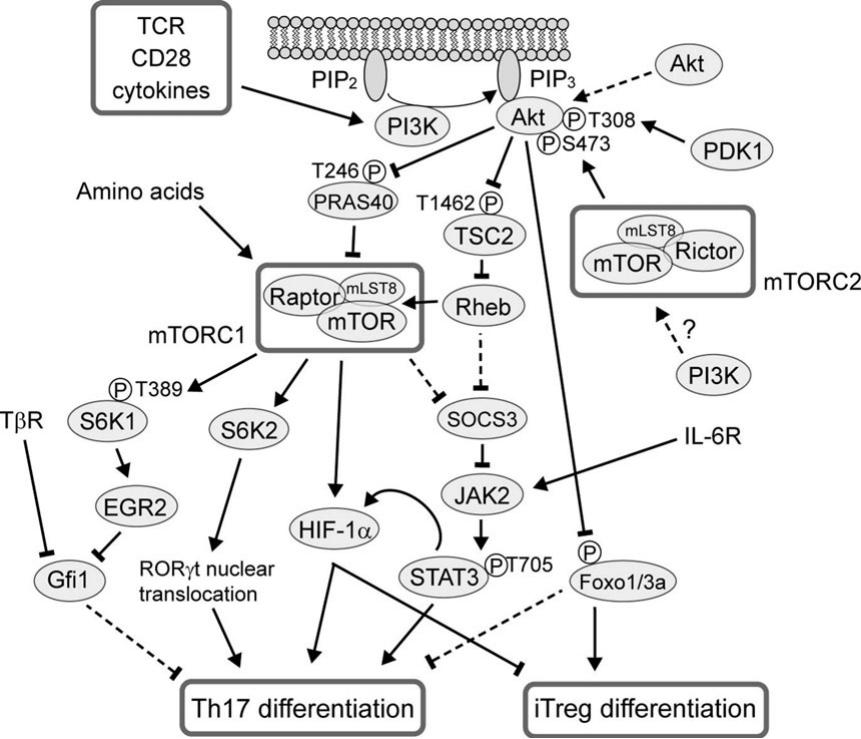
PI3K, Akt and mTOR complexes regulate Th17 differentiation. Stimulation of TCR, CD28 and cytokine receptors activates PI3K and mTORC2 in CD4^+^ T cells. PI3K activation enables Thr308 phosphorylation of Akt by PDK1 and Ser473 phosphorylation of Akt by mTORC2. TSC2 and PRAS40 are negative regulators of mTORC1 activity, and Thr308-phosphorylated Akt phosphorylates and inactivates these molecules. Activated mTORC1 enhances HIF-1α expression and RORγt nuclear translocation, whereas it negatively regulates the expression of Gfi1 and SOCS3, both of which are negative regulators of Th17 differentiation. Ser473-phosphorylated Akt also phosphorylates and inactivates Foxo1 and Foxo3a, both of which limit CD4^+^ T-cell activation and are suggested as negative regulators of Th17 differentiation.

Antigen presentation also leads to the activation of PI3K and mTORC2 in CD4^+^ T cells (Kurebayashi *et al*. 2012; Okkenhaug *et al*. 2006; also see Fig. 3). PI3K phosphorylates the third position of the hydroxyl group in the inositol ring of phosphatidylinositol and generates PIP_3_, which recruits a constitutively active kinase PDK1 and its substrate Akt to the cell membrane through interaction with their PH domains. PDK1 then phosphorylates Akt at Thr308 (Koyasu 2003). The expression of p110δ, a catalytic subunit of the class I_A_ PI3K family, is restricted to lymphocytes. Replacement of wild-type p110δ with a kinase-defective p110δ^D910A^, inhibition of p110δ by a specific inhibitor (IC87114), or deletion of p85α, a regulatory subunit that forms a heterodimer with p110δ, severely impair the phosphorylation of Akt at Thr308 upon TCR stimulation (Okkenhaug *et al*. 2006; Shiroki *et al*. 2007; Kurebayashi *et al*. 2012). There are two mTOR complexes: one is the rapamycin-sensitive mTORC1 composed of mTOR, mLST8 and Raptor, and the other is the rapamycin-insensitive mTORC2 composed of mTOR, mLST8 and Rictor (Wullschleger *et al*. 2006; also see Fig. 3). Among these, mTORC2 is able to phosphorylate Akt at Ser473 in TCR-stimulated CD4^+^ T cells (Lee *et al*. 2010). Although it is still poorly understood how mTORC2 is activated and phosphorylates Akt, studies have shown that this reaction requires the trafficking of Akt to the cell membrane upon PI3K activation (Andjelkovic *et al*. 1997). Therefore, the pan-PI3K inhibitor LY294002 also inhibits Ser473 phosphorylation of Akt. Interestingly, Thr308-phosphorylated Akt and Ser473-phosphorylated Akt phosphorylate distinct substrates; Thr308-phosphorylated Akt activates mTORC1 and enhances S6K1 phosphorylation, whereas Ser473-phosphorylated Akt preferentially phosphorylates Foxo1 and Foxo3a (Jacinto *et al*. 2006). Therefore, there are two major pathways crossing at Akt: one is the PI3K-Akt(pThr308)-mTORC1 signaling and the other is the mTORC2-Akt(pSer473)-Foxo1/3a signaling, and these demarcations are used in this review for convenience.

The role of mTORC2-Akt-Foxo1/3a signaling is well characterized in the differentiation of both nTreg and iTreg cells. Foxo1 and Foxo3a directly bind to *Foxp3* promoter and increase the expression of *Foxp3*, and the deletion of Foxo results in the collapse of T-cell homeostasis and in the severe autoimmunity (Harada *et al*. 2010; Ouyang *et al*. 2010). Foxo proteins are active in a dephosphorylated state, and phosphorylation by Akt results in the retention of Foxo proteins in the cytoplasm by 14-3-3 proteins; hence, constitutively active Akt impairs Treg development (Haxhinasto *et al*. 2008). In contrast, inhibition of PI3K by LY294002 increases iTreg differentiation in a Foxo1/3a-dependent manner (Sauer *et al*. 2008; Harada *et al*. 2010), and the deletion of *Rictor*, encoding a central component of mTORC2, also increases iTreg differentiation (Lee *et al*. 2010).

However, the contribution of mTORC2-Akt-Foxo1/3a signaling in Th17 differentiation is still controversial. The expression of a constitutively active form of Akt enhances the differentiation of all Th subsets including Th17 cells (Arimura *et al*. 2004; Kurebayashi *et al*. 2012), and T-cell-specific deletion of Foxo1 and Foxo3a results in the autoimmunity with an increased Th17 differentiation *in vivo* (Ouyang *et al*. 2010). It is also shown that the ectopic expression of Foxo inhibits IL-17A production by human CCR6^+^CD4^+^ T cells (Wan *et al*. 2010). All of these observations seem to indicate the contribution of mTORC2-Akt-Foxo1/3a signaling to Th17 differentiation; however, the deletion of *Rictor* does not influence on the differentiation of Th17 cells (Lee *et al*. 2010; Delgoffe *et al*. 2011).

## PI3K-Akt-mTORC1 signaling in Th17 and Treg differentiation

The activation of PI3K and phosphorylation of Akt at Thr308 lead to the phosphorylation and inhibition of TSC1/TSC2 complex and PRAS40 (Jacinto *et al*. 2006; Wullschleger *et al*. 2006; Laplante & Sabatini 2012). TSC1/TSC2 complex negatively regulates mTORC1 activity by impairing Rheb GTPase activity, which is required for mTORC1 activation. In addition, PRAS40 directly associates with mTORC1 and down-regulates its activity. Extracellular amino acids also activate mTORC1 via Ragulator–Rag complexes. Rapamycin inhibits mTORC1 with high specificity, and mTORC1 inhibitors are applied to the chemotherapies against several types of cancers and for immune suppression in organ transplantation.

In CD4^+^ T cells, the inhibition of PI3K and mTORC1 increases iTreg differentiation (Sauer *et al*. 2008). In contrast, activation of mTORC1 by IL-1β-IRAK1/4 signaling, which degrades TSC1/TSC2 complex, promotes Th17 differentiation (Gulen *et al*. 2010), and malfunction of mTORC1 by deletion of *Rheb* and *Raptor*, encoding central components of mTORC1, impairs Th17 differentiation (Delgoffe *et al*. 2009, 2011; Kurebayashi *et al*. 2012). There are several independent mechanisms supposed to regulate Th17 differentiation via mTORC1. One is the positive regulation of HIF-1α expression downstream of mTORC1 pathway (Ikejiri *et al*. 2011; Shi *et al*. 2011). As reviewed above, HIF-1α expression positively regulates Th17 differentiation by directly promoting *Rorc* and Th17-related gene expression (Dang *et al*. 2011), and it also increases glycolytic activity required for rapid T-cell expansion (Shi *et al*. 2011). We showed that mild hypoxia (5% oxygen) during Th17 differentiation induces the activation of the mTORC1 pathway independently of PI3K, implying the existence of a positive feedback loop between mTORC1 and HIF-1 for Th17 differentiation (Ikejiri *et al*. 2011).

Second, mTOR complexes differently regulate the phosphorylation of STAT proteins during Th differentiation. Delgoffe *et al*. (2009) showed that the deletion of *Flap*, which encodes mTOR, deprives cells of both mTORC1 and mTORC2 and reduces tyrosine phosphorylation of STAT transcription factors and the differentiation of all Th subsets with increased iTreg development albeit without TGF-β. They also reported that the deletion of *Rheb*, which severely impairs mTORC1 function, selectively reduces the tyrosine phosphorylation of STAT3 and STAT4 by inducing SOCS3 expression and impairs the differentiation of Th1 and Th17 cells (Delgoffe *et al*. 2011). Although the deletion of *Flap* or *Rheb* impairs tyrosine phosphorylation of STAT3, mTORC1 inhibition by rapamycin does not interfere with the serine and tyrosine phosphorylation of STAT3 (Lee *et al*. 2010; Kurebayashi *et al*. 2012). Although the deletion of *Raptor*, encoding an essential component of mTORC1, results in embryonic lethality (Guertin *et al*. 2006), tamoxifen-induced deletion of *Raptor* has enabled us to examine the role of mTORC1 in cells of interest (Hoshii *et al*. 2012). Deletion of *Raptor* in T cells impairs Th17 differentiation without affecting tyrosine phosphorylation of STAT3 or Th1 differentiation (Kurebayashi *et al*. 2012). These observed differences between *Flap*, *Rheb* and *Raptor* deficiencies in the regulation of STAT phosphorylation and Th1 differentiation indicate some unknown mTORC1-independent roles of mTOR and Rheb in STAT phosphorylation.

We have also shown that Th17 differentiation is impaired by mTORC1 inhibition via decreased RORγt nuclear translocation and increased Gfi1 expression, whereas Th1 differentiation is maintained both *in vivo* and *in vitro* (Kurebayashi *et al*. 2012). Gfi1 expression is suppressed by EGR1 and EGR2 transcription factors, which directly bind to *Gfi1* promoter (Laslo *et al*. 2006). Expression of EGR1 (Sarker & Lee 2004) and EGR2 (Carnevalli *et al*. 2011) is regulated by S6K1 downstream of mTORC1. Accordingly, we showed that forced expression of a constitutively active form of S6K1 in CD4^+^ T cells induced *Egr2* expression, suppressed *Gfi1* expression and accelerated Th17 differentiation (Kurebayashi *et al*. 2012), indicating that the PI3K-Akt-mTORC1-S6K1 pathway positively regulates IL-17 expression through the suppression of Gfi1. In addition to the suppression of Gfi1, mTORC1 accelerates the nuclear translocation of RORγt (Kurebayashi *et al*. 2012). RORγt does not have a nuclear localization signal (NLS), yet is localized in the nucleus in Th17 cells. We have shown that S6K2, a nuclear counterpart of S6K1, possesses a NLS, binds to RORγt and transports RORγt to the nucleus in a piggyback fashion. The expression of S6K2 is increased after TCR stimulation partly in a mTORC1-dependent fashion. Thus, the PI3K-Akt-mTORC1-S6K2 pathway also positively controls Th17 differentiation by nuclear translocation of RORγt (Kurebayashi *et al*. 2012).

In contrast to iTreg differentiation, nTreg development in the thymus is independent of mTORC1 activity. For example, the T-cell-specific deletion of TSC1, an inhibitory molecule of mTORC1, does not alter the size of the nTreg population *in vivo* (Yang *et al*. 2011a). Similarly, development of nTreg cells *in vivo* was little affected by the deletion of *Raptor* in T-cell lineage (S.M. & M.O. personal communication). Such difference in the susceptibility of nTreg and iTreg differentiation to the changes in mTORC1 activity is an intriguing subject for future studies.

Recently, a patient with a premature stop codon in *PIK3R1*, resulting in the absence of p85α but normal expression of p55α and p50α, has been reported (Conley *et al*. 2012). The patient shows agammaglobulinemia because of a severe defect in B-cell development in agreement with previous studies with mice lacking p85α (Suzuki *et al*. 1999, 2003). Further studies would reveal the role of PI3K in Th17 differentiation in humans.

## Role of PI3K-Akt-mTORC1 signaling and HIF-1 in autoimmunity and host defense

Recent studies have also established the roles of PI3K-Akt-mTORC1 signaling and transcription factor HIF-1 in the development of autoimmunity. Mice expressing inactive form of p110δ (p110δ^D910A^) show mild symptoms in EAE associated with decreased Th17 differentiation (Haylock-Jacobs *et al*. 2011). Although this study cannot exclude the possible contribution of PI3K in non-T cells to Th17 differentiation, adoptive transfer of p85α-deficient naive CD4^+^ T cells in murine T-cell transfer model of colitis shows decreased Th17 differentiation compared to wild-type CD4^+^ T cells, with Th1 differentiation maintained (our unpublished observations), indicating the pivotal roles of PI3K in CD4^+^ T cells in *in vivo* Th17 differentiation. Delgoffe *et al*. (2011) have shown that impaired mTORC1 function by deletion of *Rheb* in T cells also decreases *in vivo* generation of Th17 cells and attenuates the severity of EAE. Intriguingly, however, these mice presented an increased rate of ataxia because of mononuclear infiltration into the cerebellum instead of spinal cord. Similarly, the depletion of HIF-1α significantly decreases EAE severity by decreasing Th17 differentiation and increasing iTreg cells *in vivo* (Dang *et al*. 2011). Because RORγt controls GM-CSF expression in Th17 cells (Codarri *et al*. 2011), these data also implicate the role of PI3K, mTORC1 and HIF-1 in GM-CSF production from CD4^+^ T cells, a pivotal cytokine in the pathogenicity of myelin-reactive CD4^+^ T cells.

Treatment with mTORC1-specific inhibitor rapamycin in murine CD4^+^ T-cell transfer model of colitis also decreases Th17 differentiation and attenuates the decrease in body weights (Kurebayashi *et al*. 2012). This may partly be because mTORC1 inhibition decreases the expression of IL-23R on CD4^+^ T cells, a receptor for cytokine (IL-23) that is required for the induction of IL-17A/IFN-γ double-positive T cells and exacerbates the clinical course in murine colitis (Ahern *et al*. 2010). Additionally, mTORC1 function is required for T-cell proliferation and trafficking (Sinclair *et al*. 2008), which may also explain the regulatory effect of rapamycin in murine colitis. Especially, the importance of lymphopenia-driven proliferation in the development of CD4^+^ T-cell transfer model of colitis is well documented (Zhang & Bevan 2012). Notably, rapamycin treatment in CD4^+^ T-cell transfer model of colitis increases the differentiation of Th1 cells in mesenteric lymph nodes (Kurebayashi *et al*. 2012), possibly due to both T-cell intrinsic deviation to Th1 development and the increased production of IL-12 from APCs in the absence of PI3K-Akt-mTORC1 pathway (Fukao *et al*. 2002; Ohtani *et al*. 2008; Weichhart *et al*. 2008).

Compared to their function in the development of autoimmunity, the roles of PI3K-Akt-mTORC1 signaling and HIF-1 of CD4^+^ T cells in the host defense are still largely unknown, despite the major roles of PI3K-Akt-mTORC1 pathway in APCs to regulate Th1- and Th17-type immune reactions (Fukao *et al*. 2002; Ohtani *et al*. 2008; Weichhart *et al*. 2008). It has been shown that both Rheb and HIF-1α deficiency lead to the impaired Th17 differentiation in intestinal mucosa (Dang *et al*. 2011; Delgoffe *et al*. 2011). However, the contribution of PI3K and Akt in the generation of intestinal Th17 cells has not been reported. Cytokines and environmental factors that up-regulate mTORC1 activity and HIF-1 expression in the intestinal T cells are also largely unknown although inflammatory cytokines represented by IL-1β seem to be pivotal. The homeostatic differentiation and maintenance of Th17 cells in intestinal tissue mainly require IL-1β induced by microbiota (Shaw *et al*. 2012), and IL-1β is known to induce mTORC1 activation via IRAK1/4-mediated degradation of TSC1/2 complex in CD4^+^ T cells (Gulen *et al*. 2010). IL-1β is also known to induce HIF-1α expression even in a normoxic condition via PI3K and mTORC1 activation (Stiehl *et al*. 2002). TGF-β is another cytokine required for the *de novo* differentiation of intestinal Th17 cells (Ghoreschi *et al*. 2010), and TGF-β is a well-known activator of PI3K-Akt signaling (Zavadil & Bottinger 2005); hence, it is possible that TGF-β-induced activation of PI3K-Akt signaling also contributes to the differentiation and maintenance of Th17 cells in the intestinal mucosa.

Regarding the intestinal environment, the partial pressure of oxygen (pO_2_) in the capillary beds and mucosal interstitium of intestine is expected to be lower than those in pulmonary vein and alveoli, and this mildly hypoxic condition possibly supports the differentiation of Th17 cells in peripheral tissues including intestine as shown *in vitro* (Dang *et al*. 2011; Ikejiri *et al*. 2011; Shi *et al*. 2011). Immune homeostasis in the intestine is maintained in part by deprivation of essential amino acid tryptophan by IDO, and deprivation of essential amino acid potentially suppresses the activity of mTORC1 in CD4^+^ T cells as shown in several cell lines (Laplante & Sabatini 2012), which may result in the preferential suppression of Th17 differentiation. Actually, a small-molecule halofuginone selectively inhibits Th17 differentiation by activating amino acid starvation response and cytoprotective signaling pathway (Sundrud *et al*. 2009) and lower essential amino acid concentration preferentially decreases Th17 differentiation (our unpublished observations). Hence, PI3K-Akt-mTORC1 signaling and HIF-1 in CD4^+^ T cells may also contribute to the development of Th17 cells in mucosal tissues and to the maintenance of immune homeostasis with commensal microbiota.

## Conclusion

Th17 cells have established a unique position among Th subsets by regulating neutrophil-mediated immune responses, and their differentiation and function is controlled by a number of intracellular signaling pathways and a complex transcription factor network as reviewed here. Recent findings further identified mTORC1 as another positive regulator of Th17 differentiation, acting via the cooperative regulation of STAT3 phosphorylation, RORγt nuclear translocation and Gfi1 and HIF-1α expression. This accumulating evidence now provides us more precise understandings of Th17 differentiation and clues to the pharmacological manipulation. However, it is also true that there are many issues that remain unresolved. For instance, although studies indicate the existence of both conventional Th17(β) cells and more pro-inflammatory Th17(23) cells *in vivo*, the spatiotemporal regulation and its molecular mechanism in the generation of these different Th17 subsets are still largely unknown. Studies on these matters would provide us more knowledge about T cell–mediated immunity and opportunities for manipulating immune systems in many inflammatory disorders and infections.
